# Growth, ammonium metabolism, and photosynthetic properties of *Ulva australis* (Chlorophyta) under decreasing pH and ammonium enrichment

**DOI:** 10.1371/journal.pone.0188389

**Published:** 2017-11-27

**Authors:** Leah B. Reidenbach, Pamela A. Fernandez, Pablo P. Leal, Fanny Noisette, Christina M. McGraw, Andrew T. Revill, Catriona L. Hurd, Janet E. Kübler

**Affiliations:** 1 Department of Biology, California State University at Northridge, Northridge, California, United States of America; 2 Institute for Marine and Antarctic Studies, University of Tasmania, Hobart, Tasmania, Australia; 3 GEOMAR Helmholtz Centre for Ocean Research, Kiel, Germany; 4 Department of Chemistry, University of Otago, Dunedin, New Zealand; 5 CSIRO, Oceans and Atmosphere, Hobart, Tasmania, Australia; The University of Hong Kong, HONG KONG

## Abstract

The responses of macroalgae to ocean acidification could be altered by availability of macronutrients, such as ammonium (NH_4_^+^). This study determined how the opportunistic macroalga, *Ulva australis* responded to simultaneous changes in decreasing pH and NH_4_^+^ enrichment. This was investigated in a week-long growth experiment across a range of predicted future pHs with ambient and enriched NH_4_^+^ treatments followed by measurements of relative growth rates (RGR), NH_4_^+^ uptake rates and pools, total chlorophyll, and tissue carbon and nitrogen content. Rapid light curves (RLCs) were used to measure the maximum relative electron transport rate (rETR_max_) and maximum quantum yield of photosystem II (PSII) photochemistry (F_v_/F_m_). Photosynthetic capacity was derived from the RLCs and included the efficiency of light harvesting (α), slope of photoinhibition (β), and the light saturation point (E_k_). The results showed that NH_4_^+^ enrichment did not modify the effects of pH on RGRs, NH_4_^+^ uptake rates and pools, total chlorophyll, rETR_max_, α, β, F_v_/F_m_, tissue C and N, and the C:N ratio. However, E_k_ was differentially affected by pH under different NH_4_^+^ treatments. E_k_ increased with decreasing pH in the ambient NH_4_^+^ treatment, but not in the enriched NH_4_^+^ treatment. NH_4_^+^ enrichment increased RGRs, NH_4_^+^ pools, total chlorophyll, rETR_max_, α, β, F_v_/F_m_, and tissue N, and decreased NH_4_^+^ uptake rates and the C:N ratio. Decreased pH increased total chlorophyll content, rETR_max_, F_v_/F_m_, and tissue N content, and decreased the C:N ratio. Therefore, the results indicate that *U*. *australis* growth is increased with NH_4_^+^ enrichment and not with decreasing pH. While decreasing pH influenced the carbon and nitrogen metabolisms of *U*. *australis*, it did not result in changes in growth.

## Introduction

Since the industrial revolution, the atmospheric CO_2_ concentration has increased from 280 μatm to over 390 μatm, and about 30% of the additional CO_2_ has been absorbed by the ocean [1]. This results in ocean acidification, a term which describes the contemporary reduction in seawater pH by ca. 0.1 units with an expected further reduction of 0.3–0.5 units by 2100 [2–5]. In addition to ocean acidification, coastal regions receive inputs of excess nitrogen from aquaculture, agriculture, wastewater treatment, and the burning of fossil fuels [6,7]. Excess nitrogen is the commonly regarded cause for green algal blooms world-wide, and they are typically dominated by macroalgae from the genus *Ulva* [8–10]. Green algal blooms can impose negative effects on their ecosystems and local human communities by decreasing biodiversity and ecosystem services [11–14]. Elevated nutrients can modify the effects of elevated pCO_2_/decreased pH on algal physiology [15–22] because nitrogen and carbon metabolisms are linked via the process of protein synthesis [23]. In order to understand how nutrient-opportunistic macroalgae, such as *Ulva* spp. will respond to future oceanic conditions, it is important to consider the interaction of elevated nutrients with decreasing pH.

Non-calcareous macroalgae have been shown to express a range of responses to future pCO_2_/pH conditions. *Hizikia fusiforme* growth rates increased under future pCO_2_/pH conditions while maximum photosynthetic rates were unchanged [24]. Growth rates of *Gracilaria chilensis* and another *Gracilaria* sp. were enhanced by future pCO_2_/pH conditions [25]. *Gracilaria lemaneiformis* growth rates were also enhanced under future pCO_2_/pH conditions, but only at an intermediate photon flux density (PFD) (160 μM photons m^-2^ s^-1^) [26]. The growth rates of thirteen species of algae, including green, red, and brown algae, had no response to future pCO_2_/pH conditions with the exception of *Hypnea musciformis*, which exhibited negative growth rates [27]. *Ulva* spp. growth rates have been shown to increase or be unaffected by future pCO_2_/pH conditions [21,28–30]. Differences in responses to elevated pCO_2_/decreased pH may be caused in part by species specific differences or by unsuitable nutrient concentrations, temperature, and/or PFD for the seaweeds to support higher growth rates.

Carbon concentrating mechanisms (CCMs) allow macroalgae to increase CO_2_ at the site of carbon fixation and may be downregulated with elevated pCO_2_/decreased pH in *Ulva* spp. [31,32]. This has been linked to increased energy availability for nutrient uptake, protein synthesis, and growth when nutrients are not limiting [31,33,34]. Therefore, elevated pCO_2_/decreased pH might change nutrient uptake, assimilation, and storage capacity of macroalgae which utilize CCMs. For example, when CCMs were reduced with elevated pCO_2_/decreased pH in *Pyropia haitanensis*, growth rates and NO_3_^-^ uptake rates increased, and photosynthetic rates increased with the combination of elevated pCO_2_ and elevated NO_3_^-^ [35]. Further, nutrients mediated the effect of elevated pCO_2_/decreased pH on *P*. *haitanensis* by increasing growth rates and nitrate reductase activity (NRA) when grown with elevated CO_2_ and NO_3_^-^ enrichment [20]. *Ulva lactuca* photosynthetic rates and NRA were increased with elevated pCO_2_/decreased pH, but only when temperature was sufficient (25°C compared to 15°C), while NO_3_^-^ uptake rates were enhanced at both temperatures with elevated pCO_2_/decreased pH [21]. Another algal species which utilizes CCMs, *Hizikia fusiforme*, was also found to have enhanced growth rates, NRA, and nitrate uptake rates with elevated pCO_2_/decreased pH [24]. The interaction of NH_4_^+^ enrichment and elevated pCO_2_/decreased pH increased growth rates of *Ulva pertusa* [22]. These studies provide evidence that local (i.e., nutrient enrichment) and global (i.e., ocean acidification) drivers of environmental change could interact to change macroalgal growth and physiology.

*Ulva* spp. are opportunistic under eutrophic conditions [9] and have potentially increased growth rates under elevated pCO_2_ alone [15,28]. Prior studies suggest nitrogen in the form of NO_3_^-^ could have interacting effects with elevated pCO_2_/decreased pH in *Ulva* spp., but less is known regarding the effects of NH_4_^+^ as a potential interacting driver [15,16,22,32]. Typically, NH_4_^+^ is the preferred form of nitrogen for *Ulva* spp. because it requires less energy for assimilation than NO_3_^-^, as NO_3_^-^ must first be reduced via nitrate reductase activity (NRA) [36]. Although NO_3_^-^ is the most abundant and common form of dissolved inorganic nitrogen (DIN) in the ocean, increasing human population densities on coasts, land use change, and decreasing ocean pH all increase the availability of NH_4_^+^ in coastal areas [37].

To test the hypothesis that there will be an interacting effect of decreasing pH and elevated NH_4_^+^ concentrations on the growth, nutrient, and photosynthetic physiology of *Ulva australis*, a laboratory growth experiment was conducted across a range of future pCO_2_/pH conditions (total scale pH (pH_T_): 7.56–7.85) under ambient and elevated NH_4_^+^ concentrations. This was followed by measurements of RGRs, NH_4_^+^ uptake rates and pools, total chlorophyll, tissue carbon and nitrogen content, and photosynthetic characteristics of photosystem II using PAM fluorometry. Multiple components of carbon and nitrogen metabolisms were measured with the aim of describing how changes in these processes integrate at the organismal level (i.e., growth). With elevated pCO_2_/decreased pH and NH_4_^+^ enrichment *Ulva* spp. should have adequate internal supply of nitrogenous and carbon skeleton precursors and may have increased growth rates, potentially leading to increases in the severity of frequency of green tide blooms.

## Methods

### Collection and acclimation

*Ulva australis* was collected from Blackmans Bay, Tasmania, Australia (42°59’56”S 147°19’8”E) in July 2015 (Austral winter). Algae were stored in plastic zip-lock bags with seawater on ice and transported to the laboratory in a cooler within five hours of collection. *U*. *australis* was identified using morphological characteristics. All visible epiphytes were carefully removed from the surface of the blades which were then rinsed with filtered seawater. The cleaned algal samples were kept in aerated seawater at 16.6°C under 200 μmol photons m^-2^ s^-1^ (measured using a 4π Li-Cor LI-193 Spherical Quantum Sensor connected to a LI‑250A portable light meter) with a 12h:12h light dark cycle for 3 days to acclimate to experimental light and temperature conditions.

### Experimental design

Three *Ulva australis* thalli with a total fresh weight of 1.07 ± 0.02 g (mean ± SEM) were placed in 650 mL chambers filled with 600 mL of seawater that was UV-sterilized and filtered through a 1 μm-filter (Polyester Felt Filter Bags, NETCO, Hobart, Australia). Peristaltic pumps (FPU500, Omega Engineering, USA) were used to provide fresh seawater to each of the 24 growth chambers at a rate of 6–8 mL/min. The pH_T_ of seawater pumped to each tank was maintained using an automated pH control system [38]. Seawater was equilibrated using a membrane contactor (Micromodule, model 0.5X1, Membrana, USA) where the appropriate mix of N_2_ and CO_2_ gas was achieved using three pairs of mass flow controllers (MFCs) set to pH_T_s of 8.05, 7.85, and 7.65 (FMA5418A and FMA545C, Omega Engineering, USA). The flow rate of each MFC was proportional to the input voltage, which was supplied by an analog output module housed in a USB chassis (NI9264 and cDAQ-9174, National Instruments, USA) using a control system similar to that described in Bockman [39].

Each of the three MFCs were randomly assigned to four ambient NH_4_^+^ and four enriched NH_4_^+^ growth chambers for a total of 24 chambers. The pH_T_ within each culture chamber was measured every 1.5–3 hours throughout the week-long experiment, monitoring the effect of *U*. *australis* photosynthesis and respiration on seawater pH_T_. The seaweed biomass: seawater volume ratio affected the pH_T_ of the culture chambers so the average pH_T_ of each chamber was denoted by measurements of pH_T_ during the dark cycle throughout the entire experiment which resulted in a continuous range of pH_T_s (7.56–7.85) representative of future seawater pH conditions.

The ambient NH_4_^+^ concentration (n = 12) served as a control for the nutrient treatment and consisted of natural, UV-sterilized, filtered seawater. The elevated concentration of NH_4_^+^ (n = 12) was achieved using an auto-dosing peristaltic pump (Jebao DP-4) programmed to deliver 12 mL of a 1000 μM NH_4_Cl solution to growth chambers every two hours. Based on NH_4_^+^ dosing rate, the NH_4_^+^ concentration in the elevated treatment was 20 μM. However, discrete measurements of seawater NH_4_^+^ concentrations on days 0, 3, and 6 showed that the average NH_4_^+^ concentration was 0.4 ± 0.3 μM in the ambient treatment and 38.0 ± 18.6 μM the enriched treatment.

## pH_T_ and total alkalinity measurements

A syringe pump (V6 pump with valve 24090, Norgren, UK) and two 12-port rotary valves (23425 valve driver with valve 24493, Norgren, UK) were used to sample seawater directly from each growth chamber. For each spectrophotometric pH measurement, a reference spectrum was acquired after flushing 25 mL of seawater through a 1 cm flow-through quartz cuvette. A spectrum (400–800 nm) was acquired using an LED light source and a UV-Vis spectrometer (BluLoop and USB2000+, Ocean Optics, USA). A dye + seawater spectrum was then obtained after mixing 200 μL of 2 mM metacresol purple sodium salt dye (211761-10G, Sigma Aldrich, Australia) with an additional 25 mL of seawater within the syringe pump. The two spectra were used to calculate an absorbance spectrum. pH_T_ was calculated using the quadratic fits of the absorbance spectra between 429–439 nm, 573–583 nm and a background signal averaged between 750–760 nm. When compared to calculations based on a single wavelength, the quadratic fit approach leads to a three-fold improvement in measurement precision [38]. Each recorded pH_T_ was the average of four replicate measurements, which took approximately three minutes to obtain. The temperature of each sample was recorded with a PT100 temperature sensor and a high-precision data logger (PT-104, PICO Technology, UK). All instrument control, spectra manipulations, and pH_T_ calculations were done using LabVIEW 2014 (National Instruments, USA).

Total alkalinity (AT) samples were calculated from water samples collected in October 2015 using seawater from the same region (Taroona, Tasmania, Australia) as was collected in July 2015 for the experiment. AT samples were poisoned with mercuric chloride (0.02% vol/vol [40]) and were analyzed at the Australian National University, using an automatic built in-house titrator (consisting of a 5 mL Tecan syringe pump (Cavro X Calibur Pump), a Pico USB controlled pH sensor, and a TPS pH electrode). AT values were then calculated using the Gran technique [40].

### Growth rates

*Ulva australis* thalli were blotted with tissue to remove excess water and weighed before the start of the experiment and after seven days. The total weight of the three thalli from each chamber was used for the analysis. The RGR, expressed as % day^-1^, was calculated as RGR = ln(*FW*_*f*_/*FW*_*i*_) x *t*^-1^ x 100 where *FW*_*i*_ is the initial fresh weight, and *FW*_*f*_ is the final fresh weight after *t* days.

### NH_4_^+^ uptake rates

At the end of the seven-day incubation period, one of the three *Ulva australis* thalli (0.43 ± 0.03 g of FW) was removed from each chamber to an Erlenmeyer flask containing 200 mL of filtered seawater with overhead light of 200 μmol photons m^-2^ s^-1^. The seawater in each flask was obtained from the automated pH control system shortly before the start of the experiment so the seawater pH_T_ in the flasks was representative of the seawater in the chambers the algae came from. The initial NH_4_^+^ concentration of 20 μM was obtained with the addition of NH_4_Cl to ambient seawater. Flasks were placed on an orbital shaker (RATEK OM7, Victoria, Australia) set to 80 rpm and continuously stirred to induce water motion and reduce boundary layer effects [41]. A 10 mL sample of the water was taken at 0 and 30 minutes, and frozen at -20°C, until defrosted and analyzed for NH_4_^+^ concentration using a QuickChem 8500 series 2 Automated Ion Analyzer (Lachat Instrument, Loveland, USA). The uptake rate (*V*) was determined according to Pedersen [42]] using the formula *V* = [(*S*_*i*_ × *vol*_*i*_)-(*S*_*f*_ × *vol*_*f*_)]/(*t* × *FW*) where *S*_*i*_ and *S*_*f*_ are the initial and final NH_4_^+^ concentrations (μM) over a period of time (*t*), *vol* is the seawater volume in the flask and *FW* is the fresh weight (g) of the algae.

### Internal soluble NH_4_^+^ pools

The boiling water extraction method was used to determine the internal soluble NH_4_^+^ pool [43]. *Ulva australis* tissue (0.18 ± 0.01 g FW) was put in a boiling tube with 20 mL of deionized water then placed in a boiling water bath for 40 minutes. The liquid was cooled, decanted, and then filtered through a 0.45 μm Whatman filter (GF/C). This process was repeated on the same algal piece three times and the concentration of internal soluble NH_4_^+^ pools was calculated using the sum of the NH_4_^+^ concentrations of the three water samples of each algal piece. NH_4_^+^ concentrations were measured as stated above.

### Photosynthetic pigments

Following the experiment, a 0.04 ± 0.001 g FW piece of *Ulva australis* from each experimental chamber was kept at -20°C pending analysis. Each sample was then ground in 5 mL of 100% ethanol with a ceramic mortar and pestle in dim light and with the samples shaded. The extract was poured into 10 mL centrifuge tubes and placed in the dark at 4°C for six hours. Samples were then centrifuged for 10 min at 4000 rpm at 4°C. Total Chl *a* and *b* concentrations in the supernatant were determined according to the quadrichroic formula from Ritchie [44] using a spectrophotometer (S-22 UV/Vis, Boeco, Germany).

### Rapid light curves

Chlorophyll fluorescence of photosystem II was measured using a Pulse Amplitude Modulation fluorometer (diving-PAM, Walz, Germany) to generate rapid light curves and obtain measurements of the maximum quantum yield of PSII photochemistry (F_v_/F_m_), which is used as an indicator of stress [45]. On day seven of the experiment, one thallus from each chamber was dark adapted for 20 minutes before exposure to a flash of saturating light to obtain maximum fluorescence (F_m_). Then a rapid light curve was generated by increasing exposure to photosynthetic active radiation (PAR) ranging from 0–422 μM photons m^-2^ s^-1^. F_v_/F_m_ was calculated by the equation F_m_-F_0_/F_m_, were F_0_ is the fluorescence under measuring light conditions (ca. 0.15 μmol photons m^-2^ s^-1^) and F_m_ is the maximum fluorescence under saturating light conditions. Relative ETR (rETR) was calculated by the equation rETR = Y * PAR * 0.5. A hyperbolic curve was fit to the rETRs generated by each rapid light curve using a modified equation of Walsby [46]:

$${rETR}_{c}={rETR}_{max}(1-\exp\left(-\frac{\alpha I}{P_{m}})\right)+\beta I$$

where rETR_c_ is the calculated rETR, rETR_max_ is the maximum ETR at light saturating PFDs, α is the initial slope of the curve during light-limiting PFDs, and β is the slope of photoinhibition at high PFDs. The coefficients used in the equation were calculated using a least squares method [46].

### Total carbon and nitrogen content

A 0.35 ± 0.03 g FW section was dried at 60°C overnight, ground to a fine powder, and then analyzed for total tissue carbon and nitrogen content. Samples were weighed into pressed tin capsules (5x8 mm, 0.2 mg; Sercon, U.K.). Carbon and nitrogen content were determined using a Fisons NA1500 elemental analyzer coupled to a Thermo Scientific Delta V Plus via a Conflo IV. Combustion and reduction were achieved at 1020°C and 650°C respectively. Percent C and N composition was calculated by comparison of mass spectrometer peak areas to those of standards with known concentrations.

### Data analysis

An analysis of covariance (ANCOVA) was used to test for the interacting effect of pH and NH_4_^+^ on physiological responses of *U*. *australis*. pH_T_ was used as the continuous factor (i.e., the covariate) and NH_4_^+^ was used as the categorical variable. The relationships of each physiological response with decreasing pH in both ambient and enriched NH_4_^+^ treatments were compared to determine if the NH_4_^+^ treatment (ambient or enriched) altered the effect of decreased pH_T_. First, the interacting term was tested to determine if the slopes of the NH_4_^+^ treatments were equal. The interaction term was dropped from the ANCOVA model if the slopes were equal (i.e., p > 0.05 for the interaction term) to test for the effects of increased pCO_2_/decreased pH and NH_4_^+^ enrichment. Outliers greater than 3 standard deviations from the mean were removed *a priori* and are indicated in the figures. ANCOVA assumptions were checked using a Shapiro-Wilk test of normality and Cochran’s Q test for homogeneity of variances. Tissue N and NH_4_^+^ pools were log transformed to meet assumption of normality. Statistical analyses were done using the statistical software R studio.

## Results

### Total pH and seawater carbonate parameters

**Fig 1:**
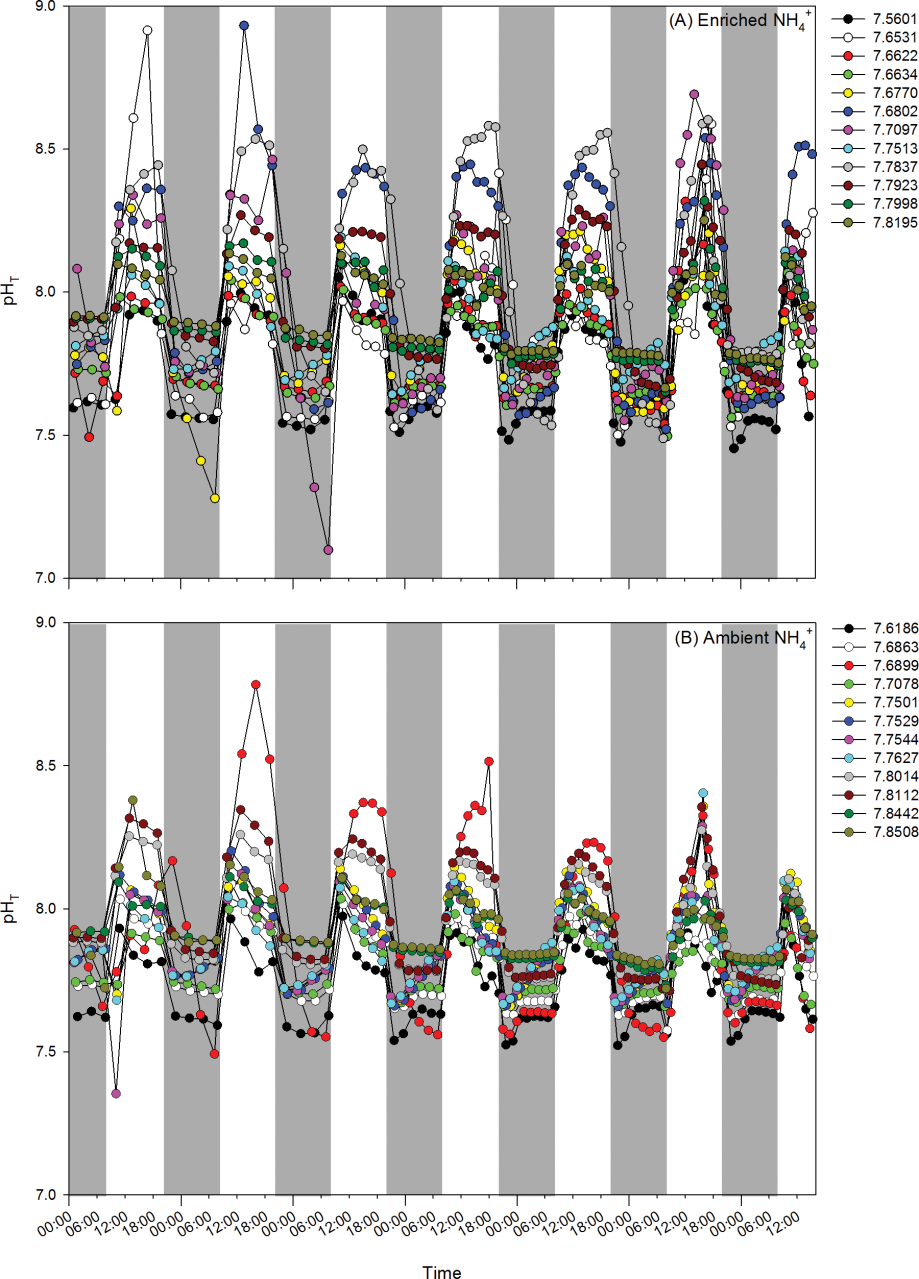
**Seven day pH**_**T**_ **regime for each chamber for** (**A) enriched NH**_**4**_^**+**^ **treatments (n = 12) and (B) ambient NH**_**4**_^**+**^ **treatments (n = 12).** The pH monitoring system took pH_T_ measurements of each *U*. *australis* growth chambers every 1.5–3 hours. Shaded areas of the graph represent dark periods.

The pH_T_ given for each treatment is the average value from the dark cycle pH_T_ measurements in each culture chamber. The measurements oscillated around the gas mixers’ set points due to algal metabolism: during the light period pCO_2_ decreased, increasing pH_T_; during the dark period cellular respiration produced CO_2_, decreasing pH_T_, with pH_T_ being relatively stable throughout the dark cycle (Fig 1). Dark cycle pH_T_ values were correlated to light and whole cycle pH_T_ values (Pearson correlation: r = 0.70, p = 0.0002 and r = 0.92, p <0.0001, respectively). Mean values for each chamber during light, dark, and whole day cycles throughout the experiment are reported in Table 1. Seawater carbonate parameters are described in the S1 Table.

**Table 1:** Mean and confidence intervals (CI: Mean [H+] ± (-log (SEM of [H+]))) for pH_T_ of each chamber for light, dark, and whole day cycles. n = number of samples collected during cycle throughout experiment.

MFC	NH_4_^+^	Light Cycle pH_T_	n	Dark Cycle pH_T_*	n	Whole Day pH_T_	n
1	Ambient	7.92 (7.88, 7.96)	50	7.69 (7.67, 7.71)	39	7.81 (7.79, 7.84)	92
1	Ambient	7.82 (7.81, 7.84)	50	7.71 (7.70, 7.71)	36	7.78 (7.77, 7.79)	92
1	Ambient	7.77 (7.75, 7.78)	50	7.62 (7.61, 7.63)	39	7.70 (7.69, 7.71)	92
1	Ambient	7.85 (7.83, 7.86)	50	7.69 (7.68, 7.69)	38	7.78 (7.76, 7.79)	92
1	Enriched	7.83 (7.80, 7.85)	50	7.56 (7.55, 7.57)	39	7.69 (7.68, 7.71)	92
1	Enriched	7.84 (7.81, 7.86)	50	7.66 (7.65, 7.67)	38	7.76 (7.74 7.78)	91
1	Enriched	7.85 (7.82, 7.88)	50	7.65 (7.63, 7.67)	38	7.76 (7.74, 7.78)	92
1	Enriched	7.85 (7.83, 7.86)	50	7.66 (7.65, 7.67)	40	7.76 (7.74, 7.77)	92
2	Ambient	7.92 (7.89, 7.95)	48	7.75 (7.75, 7.76)	37	7.84 (7.83, 7.86)	90
2	Ambient	7.93 (7.91, 7.95)	49	7.76 (7.75, 7.77)	37	7.85 (7.84, 7.86)	91
2	Ambient	7.95 (7.93, 7.98)	49	7.75 (7.74, 7.76)	35	7.86 (7.85, 7.88)	91
2	Ambient	7.95 (7.94, 7.97)	46	7.75 (7.74, 7.76)	38	7.86 (7.84, 7.87)	87
2	Enriched	7.92 (7.87, 7.96)	49	7.68 (7.66, 7.69)	35	7.81 (7.78, 7.83)	91
2	Enriched	7.93 (7.90, 7.97)	50	7.75 (7.74, 7.76)	36	7.85 (7.83,7.87)	91
2	Enriched	8.09 (8.04, 8.15	49	7.68 (7.66, 7.70)	37	7.88 (7.85,7.91)	91
2	Enriched	7.97 (7.91, 8.04)	50	7.71 (7.68, 7.74)	38	7.85 (7.81, 7.88)	91
3	Ambient	8.04 (8.01, 8.06)	50	7.80 (7.79, 7.81)	39	7.92 (7.91, 7.94)	92
3	Ambient	8.05 (8.03. 8.08)	50	7.81 (7.80, 7.82)	39	7.93 (7.92, 7.95)	92
3	Ambient	7.97 (7.95, 7.98)	51	7.84 (7.84, 7.85)	36	7.92 (7.91, 7.92)	91
3	Ambient	7.97 (7.96, 7.99)	50	7.85 (7.85, 7.86)	36	7.92 (7.91, 7.93)	92
3	Enriched	8.06 (8.03, 8.09)	50	7.79 (7.78, 7.81)	38	7.93 (7.91, 7.95)	92
3	Enriched	8.07 (8.02, 8.12)	50	7.78 (7.75, 7.81)	37	7.94 (7.91, 7.97)	92
3	Enriched	7.99 (7.98, 8.01)	51	7.82 (7.81, 7.83)	36	7.92 (7.90, 7.93)	91
3	Enriched	8.00 (7.98, 8.02)	51	7.80 (7.79, 7.81)	36	7.91 (7.90, 7.93)	91

* The dark cycle pH_T_ averages were calculated throughout the last 11 hours of the dark cycle.

### Interactive effects

The slopes for all dependent variables, with the exception of E_k_, were indistinguishable between ambient and enriched NH_4_^+^ treatments as indicated by the non-significant interaction terms (pH_T_ and NH_4_^+^) in the ANCOVAs (Table 2). The following results of those dependent variables with a non-significant interaction are reported as ANCOVAs with the interacting term dropped from the model.

**Table 2:** ANCOVA results for *Ulva australis*.

		Full Model^a^				Partial Model^b^			
Variable	Source of	Degrees of	F-value	p-value*	Model R^2^	Degrees of	F-value	p-value*	Model R^2^
	Variation	Freedom			(p-value)*	Freedom			(p-value)*
RGR	pH	1	2.0011	0.1726	0.571	1	2.0900	0.1630	0.5687
	NH_4_^+^	1	24.5112	0.0001	(**<0.0001**)	1	25.6000	**0.0001**	(**<0.0001**)
	pH x NH_4_^+^	1	0.1070	0.7470					
	Residuals	20				21			
NH_4_^+^	pH	1	0.8715	0.3617	0.3195	1	0.9148	0.3497	0.3193
uptake	NH_4_^+^	1	8.5138	0.0085	(**0.0485**)	1	8.9374	**0.0070**	(**0.01761**)
rates	pH x NH_4_^+^	1	0.0048	0.9455					
	Residuals	20				21			
NH_4_^+^	pH	1	0.0007	0.9794	0.4094	1	0.0007	0.9789	0.4061
pools	NH_4_^+^	1	13.0640	0.0018	(**0.0165**)	1	13.6771	**0.0014**	** **(**0.0055**)
	pH x NH_4_^+^	1	0.1035	0.7511					
	Residuals	19				20			
Total	pH	1	6.7120	0.0175	0.4865	1	7.0470	**0.0148**	0.4864
Chl	NH_4_^+^	1	12.2325	0.0023	(**0.0034**)	1	12.8430	**0.0018**	** **(**0.0009**)
	pH x NH_4_^+^	1	0.0018	0.9669					
	Residuals	20				21			
rETR_max_	pH	1	11.9689	0.0025	0.7062	1	12.4760	**0.0020**	0.704
	NH_4_^+^	1	35.9519	<0.0001	(**<0.0001**)	1	37.4740	**<0.0001**	(**<0.0001**)
	pH x NH_4_^+^	1	0.1473	0.7052					
	Residuals	20				21			
E_k_	pH	1	4.4425	0.0479	0.3164	—	—	—	—
	NH_4_^+^	1	0.8463	0.0385	(0.0506)	—	—	—	
	pH x NH_4_^+^	1	4.7757	**0.0409**					
	Residuals	20				—			
α	pH	1	0.0001	0.9936	0.3342	1	0.0001	0.9938	0.2565
	NH_4_^+^	1	7.7059	0.0117	(**0.0397**)	1	7.2452	**0.0137**	(**0.0445**)
	pH x NH_4_^+^	1	2.3354	0.1421					
	Residuals	20				21			
β	pH	1	0.0215	0.8850	0.4442	1	0.0195	0.8902	0.3581
	NH_4_^+^	1	12.8634	0.0018	(**0.0073**)	1	11.6938	**0.0026**	(**0.0095**)
	pH x NH_4_^+^	1	3.1003	0.0936					
	Residuals	20				21			
F_v_/F_m_	pH	1	10.4249	0.0042	0.6712	1	10.5410	**0.0039**	0.6586
	NH_4_^+^	1	29.6389	<0.0001	(**<0.0001**)	1	29.9680	**<0.0001**	(**<0.0001**)
	pH x NH_4_^+^	1	0.7693	0.3909					
	Residuals	20				21			
%N	pH	1	5.7027	0.0269	0.7761	1	5.6892	**0.0266**	0.7643
	NH_4_^+^	1	62.5572	<0.0001	(**<0.0001**)	1	62.4082	**<0.0001**	(**<0.0001**)** **
	pH x NH_4_^+^	1	1.0501	0.3177					
	Residuals	20				21			
%C	pH	1	0.5404	0.4708	0.1021	1	0.5377	0.4715	0.05263
	NH_4_^+^	1	0.6318	0.4360	(0.5307)	1	0.6288	0.4367	(0.5669)
	pH x NH_4_^+^	1	1.1022	0.3063					
	Residuals	20				21			
C:N	pH	1	6.8442	0.0165	0.793	1	6.9056	**0.0157**	0.7846
	NH_4_^+^	1	68.9589	0.0000	(**<0.0001**)	1	69.5776	**0.0000**	(**<0.0001**)
	pH x NH_4_^+^	1	0.8133	0.3779					
	Residuals	20				21			

RGR, relative growth rate; Chl, Chlorophyll; rETR_max_, maximum relative electron transport rate; E_K_, light saturation point; α, the efficiency of light harvesting; β, slope of photoinhibition; F_v_/F_m_, maximum quantum yield of PSII photochemistry; %N, percent tissue nitrogen; % C, percent tissue carbon; C:N, carbon to nitrogen ratio.

*p-values in bold indicate significance (α = 0.05).

^a^The full model ANCOVA included the interaction term (pH x NH_4_^+^) to test for differences in the slopes.

^b^If the interaction was non-significant, the partial model ANCOVA including only NH_4_^+^ (the categorical variable) and the covariate pH (the continuous variable) as factors was used.

### Growth rates

**Fig 2:**
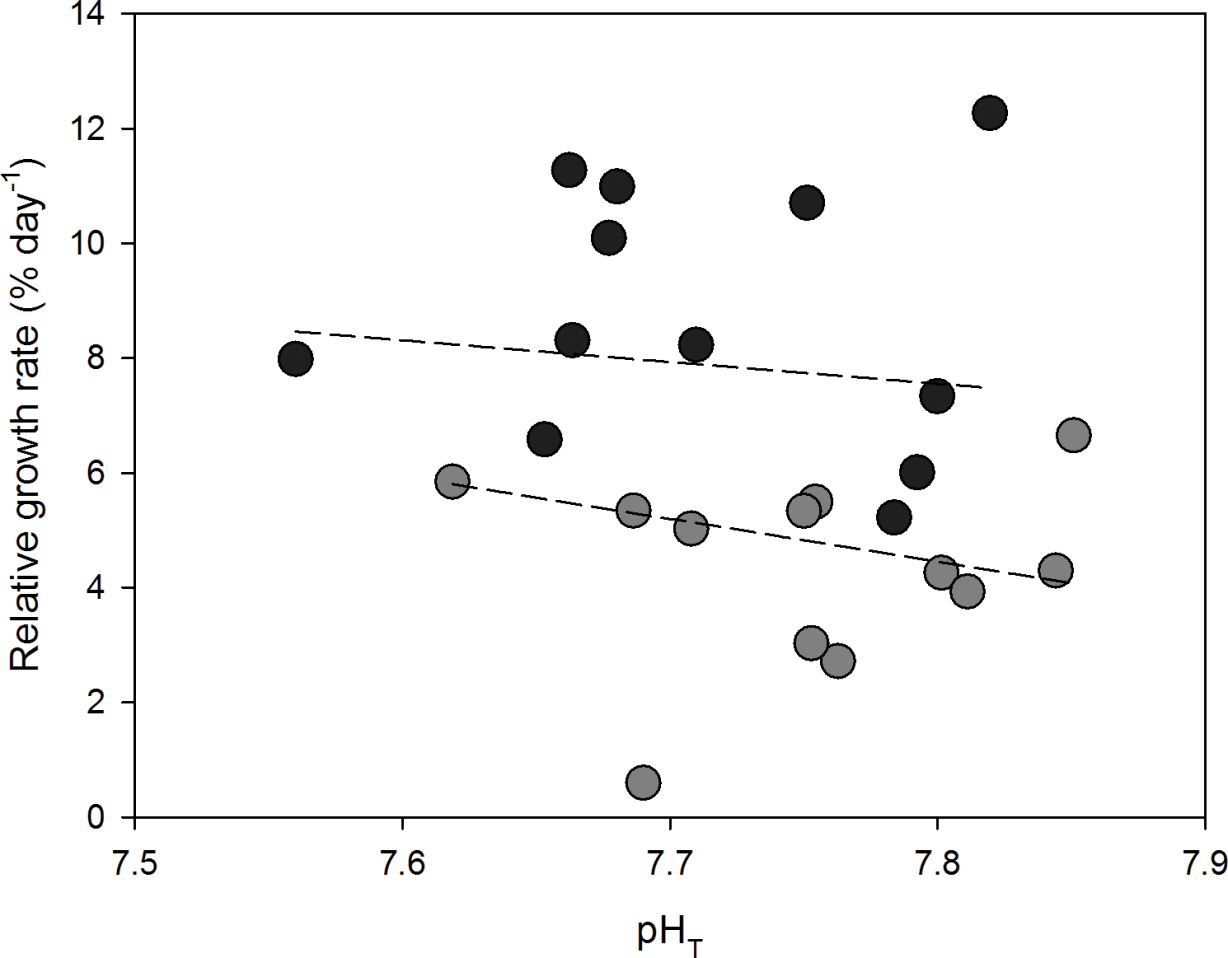
Relative growth rates (% day^-1^) for *Ulva australis* under ambient and enriched NH_4_^+^ treatments across a range of pH_T_. Grey points represent ambient NH_4_^+^ treatments and black points represent enriched NH_4_^+^ treatments. The slope of RGR with decreasing pH_T_ for each NH_4_^+^ treatment (dashed lines) were tested for an interaction using an ANCOVA.

RGRs of *Ulva australis* in enriched NH_4_^+^ treatments (8.75 ± 0.69% day^-1^, mean ± SEM) were approximately double those in the ambient NH_4_^+^ treatments (4.36 ± 0.5% day^-1^) (ANCOVA; F_1, 21_ = 25.60, p < 0.001, Fig 2). RGRs did not differ across pH_T_ treatments (ANCOVA; F_1, 21_ = 2.09, p = 0.1630).

### NH_4_^+^ uptake rates

**Fig 3:**
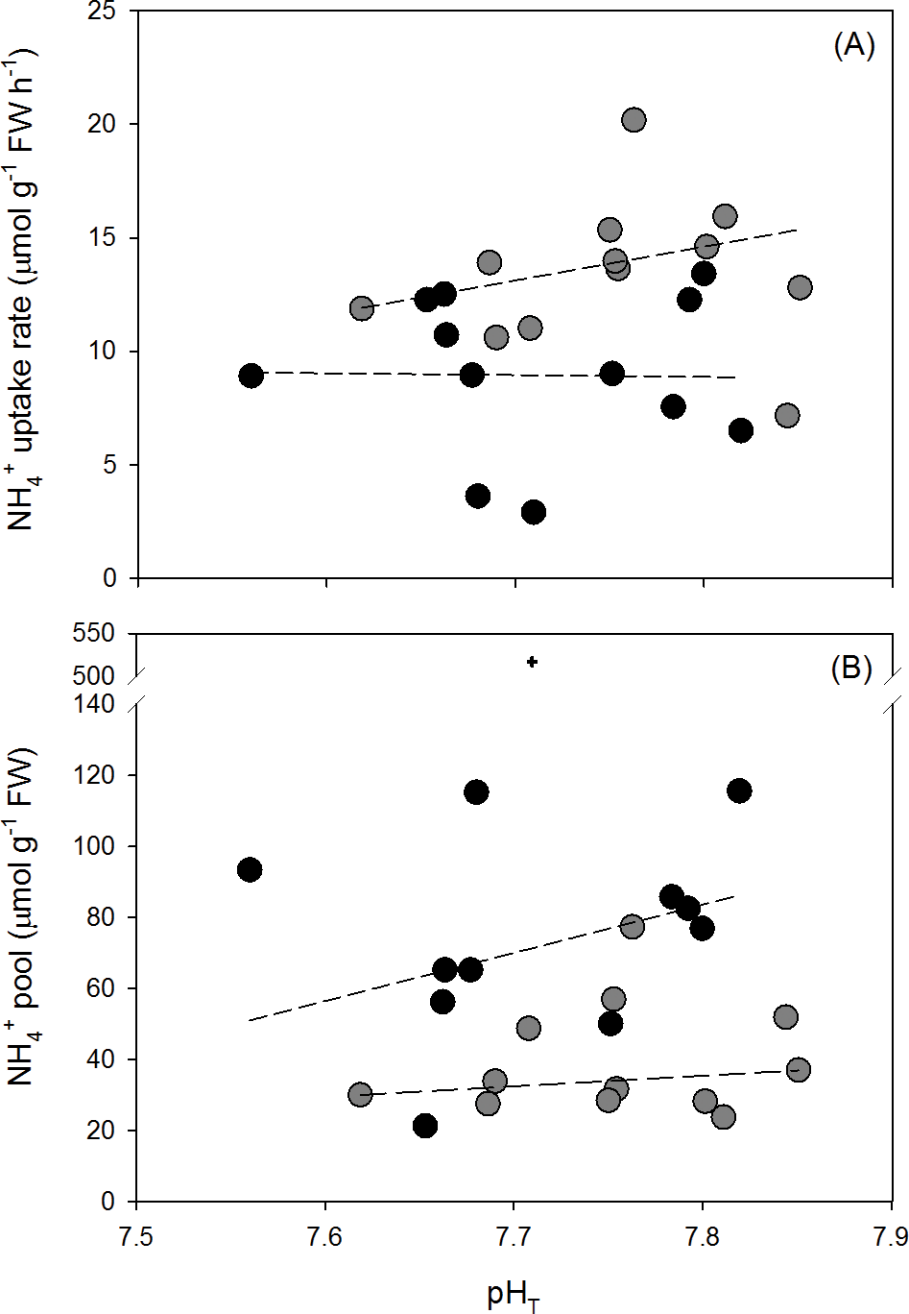
**(A) NH**_**4**_^**+**^ **uptake rates (μmol g**^**-1**^ **FW hour**^**-1**^**) in 20** μ**M NH**_**4**_^**+**^ **seawater for 30 minutes and (B) internal NH**_**4**_^**+**^ **pools (μmol g**^**-1**^ **FW) for *Ulva australis* grown under ambient and enriched NH**_**4**_^**+**^ **treatments across a range of pH**_**T**_**s.** Grey points represent ambient NH_4_^+^ treatments and black points represent enriched NH_4_^+^ treatments. A plus symbol (+) indicates an outlier which was removed for statistical analysis. The slopes of NH_4_^+^ uptake rates and internal NH_4_^+^ pools with pH_T_ for each NH_4_^+^ treatment (dashed lines) were tested for an interaction using an ANCOVA.

NH_4_^+^ uptake rates were higher in *Ulva australis* from the enriched NH_4_^+^ treatment (9.06±1.04 μmol NH_4_^+^ g^-1^ FW hour^-1^) than in the ambient NH_4_^+^ treatment (13.42±0.97 μmol NH_4_^+^ g^-1^ FW hour^-1^) (ANCOVA; F_1, 21_ = 8.9374, p = 0.007, Fig 3A). pH_T_ had no significant effect on the NH_4_^+^ uptake rates (ANCOVA; F_1, 21_ = 0.9148, p = 0.3497).

### Internal NH_4_^+^ pools

Internal NH_4_^+^ pools in *Ulva australis* thalli were higher in the enriched NH_4_^+^ treatments (75.21 ± 8.85 μmol NH_4_^+^ g^-1^ FW) than in the ambient NH_4_^+^ treatment (39.60 ± 4.81 μmol NH_4_^+^ g^-1^ FW) (ANCOVA; F_1, 20_ = 13.6771, p = 0.0041, Fig 3B). pH_T_ had no effect on the NH_4_^+^ pools (ANCOVA; F_1, 20_ = 0.0007, p = 0.9789).

### Photosynthetic pigments

**Fig 4:**
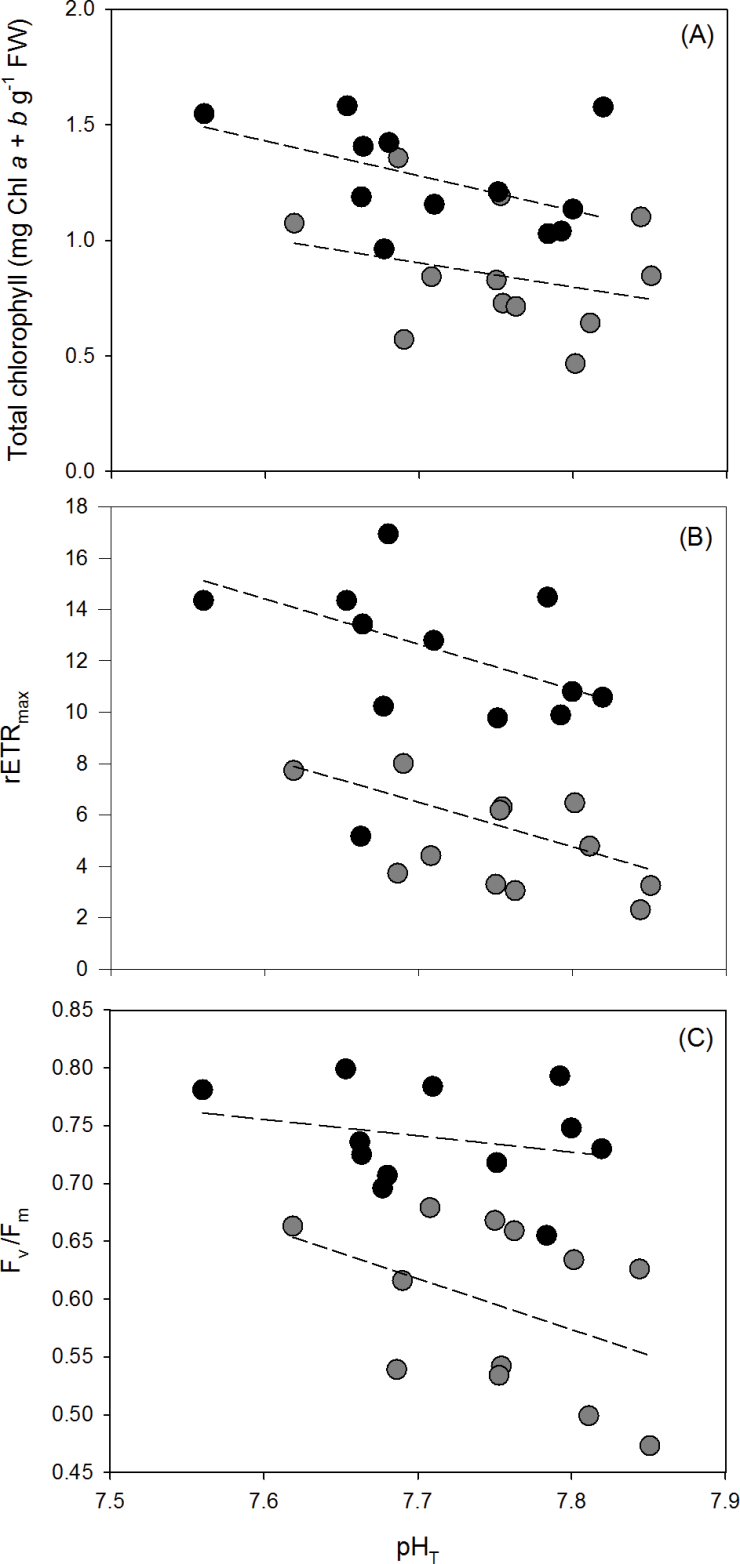
**(A) Total chlorophyll (mg Chl *a* + *b* g**^**-1**^ **FW), (B) rETR**_**max**_ **from rapid light curves, and (C) F**_**v**_**/F**_**m**_ **from rapid light curves for *Ulva australis* grown under ambient and enriched NH**_**4**_^**+**^ **treatments across a range of pH**_**T**_. Grey points represent ambient NH_4_^+^ treatments and black points represent enriched NH_4_^+^ treatments. The slopes of total chlorophyll, rETR_max_, and F_v_/F_m_ with decreasing pH_T_ for each NH_4_^+^ treatment (dashed lines) were tested for an interaction using an ANCOVA.

The total chlorophyll concentration (Chl *a + b*) content was higher in *Ulva australis* from enriched NH_4_^+^ treatments (1.27±0.07 mg g^-1^ FW) compared to the ambient NH_4_^+^ treatment (0.86±0.08 mg g^-1^ FW) (ANCOVA; F_1, 21_ = 12.8430, p = 0.0018, Fig 4A). The total chlorophyll concentration also increased with decreasing pH_T_ (ANCOVA; F_1, 21_ = 7.0470, p = 0.0148).

### Rapid light curves

rETR_max_ increased with NH_4_^+^ enrichment (ANCOVA; F_1, 21_ = 37.4740, p<0.001, Fig 4B) with an average rETR_max_ of 4.96±0.58 in the ambient NH_4_^+^ treatment and 11.9±0.94 in the enriched NH_4_^+^ treatment. rETR_max_ increased with decreasing pH (ANCOVA; F_1, 21_ = 12.4760, p = 0.0020). Like rETR_max_, the average F_v_/F_m_ was higher with NH_4_^+^ enrichment and decreasing pH (ANCOVA; F_1, 21_ = 29.9680, p<0.001 and ANCOVA; F_1, 21_ = 10.5410, p = 0.0039, respectively, Fig 4C). The F_v_/F_m_ in the ambient NH_4_^+^ treatment was 0.59±0.22 and 0.74 ± 0.01 in the enriched NH_4_^+^ treatment.

**Fig 5:**
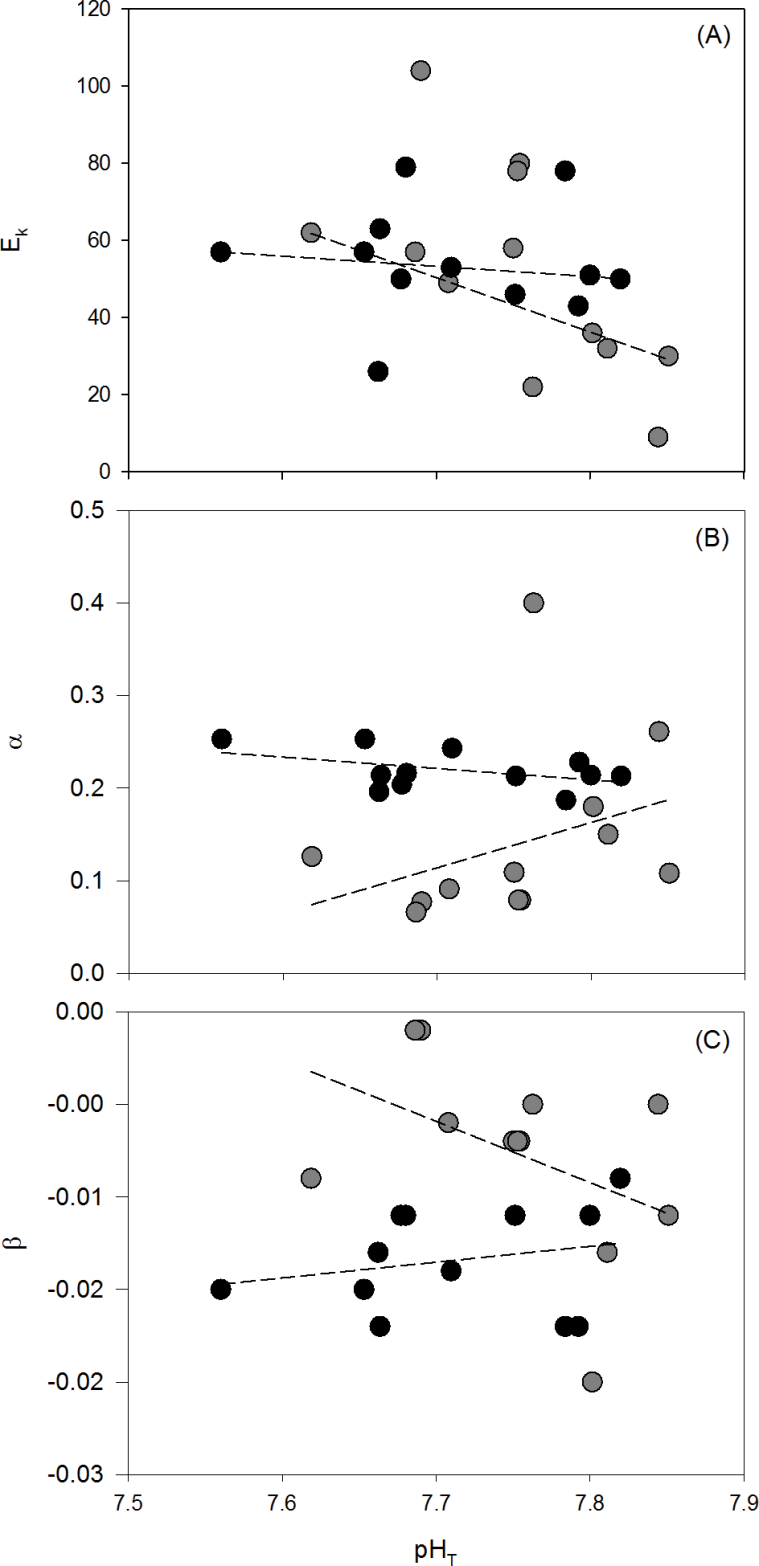
**(A) Light saturation point (E**_**k**_**), (B) initial slope of the curve (**α**), and (C) slope of photoinhibition at high photon flux densities (**β**) from rapid light curves for *Ulva australis* grown under ambient and enriched NH**_**4**_^**+**^ **treatments across a range of pH**_**T**_. Grey points represent ambient NH_4_^+^ treatments and black points represent enriched NH_4_^+^ treatments. The slopes of E_k_, α, and β with decreasing pH_T_ for each NH_4_^+^ treatment (dashed lines) were tested for an interaction using an ANCOVA.

The effect of pH on E_k_ differed between NH_4_^+^ treatments (ANCOVA; F_1, 20_ = 4.7757, p = 0.00409, Fig 5A), increasing with decreasing pH in the ambient NH_4_^+^ treatment, but not the enriched NH_4_^+^ treatment where there was no relationship between pH and E_k_. α was not influenced by pH_T_ (ANCOVA; F_1, 21_ = 0.0001, p = 0.9938, Fig 5B). However, α was greater with NH_4_^+^ enrichment (ANCOVA; F_1, 21_ = 7.2451, p = 0.0137) with a mean of 0.14±0.03 in the ambient NH_4_^+^ treatment and a mean of 0.22±0.01 in the enriched NH_4_^+^ treatment. Likewise, β was not influenced by pH_T_ (ANCOVA; F_1, 21_ = 0.0195, p = 0.8902, Fig 5C) but β was more negative in the enriched NH_4_^+^ treatments, averaging -0.008±1.58x10^-3^ in the ambient NH_4_^+^ treatment and -0.0013±8.48x10^-4^ in the enriched NH_4_^+^ treatment (ANCOVA; F_1, 21_ = 11.6938, p = 0.0026).

### Tissue carbon and nitrogen

**Fig 6:**
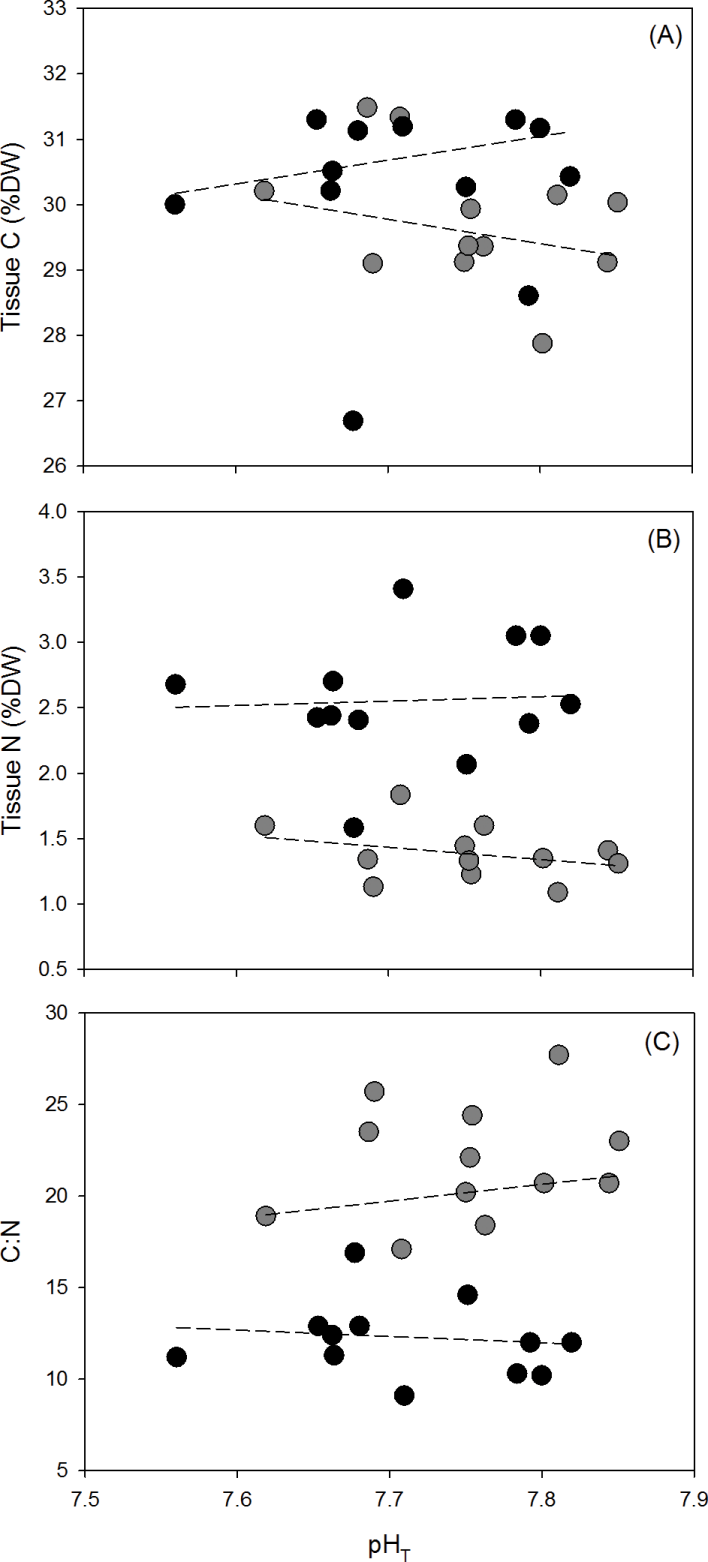
**(A) Tissue C (%DW), (B) tissue N (%DW), and (C) C:N ratio of samples of *Ulva australis* under ambient and enriched NH**_**4**_^**+**^ **treatments across a range of pH**_**T**_. Grey points represent ambient NH_4_^+^ treatments and black points represent enriched NH_4_^+^ treatments. The slopes of tissue C, tissue N, and the C:N ratio with decreasing pH_T_ for each NH_4_^+^ treatment (dashed lines) were tested for an interaction using an ANCOVA.

Tissue C (% DW) was not affected by pH or NH_4_^+^ enrichment (ANCOVA; F_1, 21_ = 0.5377, p = 0.4715 and F_1, 21_ = 0.6288 p = 0.4367, respectively) (Fig 6A). Tissue N (%DW) averaged 1.39±0.06 in the ambient NH_4_^+^ treatment and was significantly greater in the enriched NH_4_^+^ treatment with an average of 2.56±0.14 (ANCOVA; F_1, 21_ = 62.4082, p = <0.001)(Fig 6B) and increased as pH decreased (ANOVA; F_1, 21_ = 5.6892, p = 0.0266). The C:N ratio was lower in enriched NH_4_^+^ treatment with an average of 11.3±1.15, while in the ambient NH_4_^+^ treatment the average was 21.87±0.95 (ANCOVA; F_1, 21_ = 69.5776, p = <0.001)(Fig 6C). The C:N ratio decreased with decreasing pH (ANOVA; F_1, 21_ = 6.9056, p = 0.00157).

## Discussion

The growth, nutrient, and photosynthetic physiology of *Ulva australis* with increased pCO_2_/decreased pH did not depend on the NH_4_^+^ treatment, with the exception of E_k_. This was counter to the hypothesis that NH_4_^+^ enrichment and increased pCO_2_/decreased pH would interact to change *U*. *australis* growth and physiology. This study demonstrates that *U*. *australis* growth rates are more likely to be influenced by nutrient enrichment, rather than ocean acidification, as NH_4_^+^ enrichment increased activities of PSII and NH_4_^+^ pools and ultimately increased growth rates. N-deficiency has been shown to lower the ability of *Ulva rotundata* to photoacclimate to changing light regimes and can lead to declines in rETR_max_ and α in *U*. *lactuca* [47,48]. NH_4_^+^ enrichment increased total chlorophyll concentrations, rETR_max_, F_v_/F_m_, and α increased with NH_4_^+^ enrichment indicating N-deficiency inhibited photosynthesis. Photoinhibition (β) and differences in β between NH_4_^+^ enriched and ambient treatments were small at the highest PFDs measured which suggests an increased range of PFD would be better suited for demonstrating effects on β. Nutrient enrichment increased growth and photosynthetic characteristics of *U*. *australis* which has been shown with many macroalgae [8].

In this study, decreased pH influenced photosynthetic physiology as demonstrated by total chlorophyll, rETR_max_, E_k_, and F_v_/F_m_. With pH being reduced by the addition of pCO_2_, the increase in the total dissolved inorganic carbon (DIC) concentration in seawater likely contributed in the increased activity and efficiency of PSII. However, this did not result in increased growth rates. A decoupling of the photosynthetic characteristics and growth rates is not uncommon because growth is linked to multiple components of algal metabolism, not just a single process (i.e., photosynthesis). In this experiment, this decoupling may represent a tradeoff between nitrogen resources for improved photosynthetic efficiency (higher concentration of chlorophyll) or growth (resulting in dilution of chlorophyll with cellular division). Here, it was demonstrated that *Ulva australis* grown with NH_4_^+^ enrichment was better acclimated to various pH conditions with regards to E_k_, as there was no relationship between E_k_ and pH. When grown in the ambient NH_4_^+^ treatment, E_k_ increased with increasing CO_2_/decreased pH. In future pH conditions, *U*. *australis* growing in low NH_4_^+^ seawater may be able to increase their potential habitat range to include those with higher light levels. However, given enough nutrients, light limitations would be reduced and pH would have no effect on E_k_.

The supposition that macroalgal growth rates may increase with future pCO_2_/pH conditions due to energy savings from downregulation of CCMs [33,49,50] is likely not a pervasive feature of CCM utilizing macroalgae. Enhanced growth with pCO_2_ enrichment is probably the result of the influence of light levels on CCMs [51]. Energetic constraints on carbon acquisition at low PFDs increases dependence on passive CO_2_ diffusion, while CCMS are more efficient at high PFDs [33]. When PFD is low, the carbon demands of photosynthesis can be saturated by diffusion alone and CCMs are not needed. For example, pCO_2_ enrichment only enhanced *Gracilaria lemaneiformis* growth rates at an intermediate PFD [26]. Young and Gobler [32] found that *Ulva* spp. growth rates increased with pCO_2_ enrichment but varied by season, primarily increasing only in summer months. Assuming their findings are representative of *Ulva* spp. seasonal growth dynamics in a temperate location, then the results of the current study likely represent a less productive time of year for *U*. *australis*. Considering other environmental variables such as season, temperature, and light intensity are important for building a comprehensive framework from which we can elucidate patterns of ecological relevance from laboratory studies.

NH_4_^+^ enrichment increased RGRs to approximately twice that of *Ulva australis* grown in non-enriched seawater. Increased RGR with increasing nutrient concentrations is common for *Ulva* spp. [47,52], but it is also dependent on seasonal changes in light supply and ambient nitrogen levels [53]. For example, Lapointe and Tenore [54] showed that when *Ulva fasciata* was not grown with sufficient light, the enhancement of growth with NO_3_^-^ was eliminated. Furthermore, growth rates of *Ulva lactuca* more than doubled with the addition of NH_4_^+^ or NO_3_^-^ when collected from an oligotrophic site, but an increased growth rate with nutrient enrichment was not evident when algae were collected from a nutrient enriched site [55].

In the present experiment, internal NH_4_^+^ pools and tissue N content were nearly twice as large in the NH_4_^+^ enriched treatments as in the ambient treatments, indicating light and nutrients were sufficient for nutrient assimilation and growth, while the ambient NH_4_^+^ treatments were N-limiting. In the NH_4_^+^ enriched treatment, *Ulva australis* NH_4_^+^ uptake rates were slower than in the ambient NH_4_^+^ treatments, which supports the theory that nutrient histories influence nutrient uptake capabilities by feedback inhibition as internal N pools increase [56–61]. *U*. *australis* from the NH_4_^+^ enriched treatments, were still capable of NH_4_^+^ uptake despite growth under high nutrient availability and relatively concentrated NH_4_^+^ pools. This has also been demonstrated with *Ulva expansa* and *Ulva intestinalis* with varying nutrient histories [61] and shows their ability to take up surplus nutrients under growth with low and high nutrient concentrations.

The increase in tissue N, decrease in the C:N ratio and increase in E_k_ in the ambient NH_4_^+^ treatment with decreasing pH in this experiment indicate that decreased pH may provide relief from nutrient limitation. An increase in chlorophyll content and tissue N with decreasing pH support that NH_4_^+^ was assimilated to produce nitrogenous compounds such as chlorophyll, protein, and amino acids and not stored in internal NH_4_^+^ pools during this experiment. We did not detect changes in NH_4_^+^ uptake rates with decreasing pH, which corresponds to the absence of changes in NH_4_^+^ pools and growth rates. This contrasts that findings of increased NO_3_^-^ uptake rates under future pCO_2_/pH conditions in *Ulva rigida*, *Hizikia fusifome*, and *Gracilaria* spp. [15,24,25], and increased NH_4_^+^ uptake rate future pCO_2_/pH in *Hypnea spinella* [62]. The effect of pCO_2_/pH on N uptake rate may also be sensitive to temperature, as NO_3_^-^ uptake rates in *Ulva lactuca* increased with CO_2_ enrichment at 25°C, but not 15°C [21].

Based on our results, it is unlikely NH_4_^+^ enrichment (a local-scale environmental change) will interact with ocean acidification (a global-scale environmental change), to affect *Ulva australis* growth, nutrient, and photosynthetic physiology. We were able to demonstrate that increased growth rate with NH_4_^+^ enrichment could be explained by cellular changes in NH_4_^+^ and photosynthetic physiology. However, physiological responses to pH were more complex, where *Ulva australis* growth rates did not change under future pCO_2_/pH conditions, despite the fact that rETR_max_, F_v_/F_m_, and tissue N increased. These changes in photosynthetic and nutrient physiology could potentially lead to increased growth rates in macroalgae [63]. It was also demonstrated that decreased pH may reduce nutrient limitation and increase E_k_ under low NH_4_^+^ conditions. Therefore, growth rates have the potential to increase with future pCO_2_/pH conditions under a more favorable set of environmental conditions where PFD and/or season may interact to influence *U*. *australis* growth rates in future pCO_2_/pH conditions. In summary, the concern that ocean acidification may contribute to the increasing the biomass of green-tide blooms along anthropogenically influenced coastlines world-wide is not supported, despite changes in photosynthetic and nutrient physiology that could favor increased growth. However, NH_4_^+^ enrichment significantly increased growth rates of the opportunistic macroalga *U*. *australis*. This is likely to contribute to increases in the severity of green-tide blooms in areas where land-use change and development are leading to increases in NH_4_^+^ concentrations in seawater.

## Supporting information

S1 Table Seawater carbonate chemistry estimates. Measurements of total pH (pH_T_) and total alkalinity (AT) are described in the methods. AT was measured as 2111.42 ± 18.33 (mean ± SEM) (n = 7). Salinity is assumed to be 35%. Temperature is assumed to be 16.5°C (the average temperature throughout the experiment) MFC = mass flow controller. DIC = dissolved inorganic carbon.
(PDF)
