# Supplementary material for: Growth, ammonium metabolism, and photosynthetic properties of Ulva australis (Chlorophyta) under decreasing pH and ammonium enrichment
Source: PLoS One. 2017 Nov 27;12(11):e0188389. doi: 10.1371/journal.pone.0188389 (PMC5703455; doi:10.1371/journal.pone.0188389)
Supplement: S1 Table — Measurements of total pH (pHT) and total alkalinity (AT) are described in the methods. AT was measured as 2111.42 ± 18.33 (mean ± SEM) (n = 7). Salinity is assumed to be 35%. Temperature is assumed to be 16.5°C (the average temperature throughout the experiment) MFC = mass flow controller. DIC = dissolved inorganic carbon. (PDF) [file pone.0188389.s001.pdf]

Supporting information for:

Growth, ammonium metabolism, and photosynthetic properties of *Ulva australis* (Chlorophyta) under decreasing pH and ammonium enrichment.

Reidenbach LB, Fernandez PA, Leal PP, Noisette F, McGraw CM, Revill AT, Hurd CL, Kübler JE

S1 Table. Seawater carbonate chemistry estimates. Measurements of total pH (pH_T) and total alkalinity (AT) are described in the methods. AT was measured as 2111.42 ± 18.33 (mean ± SEM) (n = 7). Salinity is assumed to be 35 ‰. Temperature is assumed to be 16.5°C (the average temperature throughout the experiment) MFC = mass flow controller. DIC = dissolved inorganic carbon.

MFC	NH_4^+ treatment	Cycle	pH_T	Salinity (‰)	Temperature (°C)	pCO_2 (µatm)	HCO_3^- (µmol/kg)	CO_3^{2-} (µmol/kg)	DIC (µmol/kg)	AT (µmol/kg)
1	Ambient	light	7.92	35	16.5	513.62	1812.52	118.64	1949.49	2111.42
1	Ambient	light	7.82	35	16.5	666.02	1866.92	97.06	1987.76	2111.42
1	Ambient	light	7.77	35	16.5	756.82	1890.75	87.61	2005.37	2111.42
1	Ambient	light	7.85	35	16.5	616.45	1851.57	103.15	1976.73	2111.42
1	Enriched	light	7.83	35	16.5	649.10	1861.90	99.06	1984.12	2111.42
1	Enriched	light	7.84	35	16.5	632.58	1856.78	101.09	1980.44	2111.42
1	Enriched	light	7.85	35	16.5	616.45	1851.57	103.15	1976.73	2111.42
1	Enriched	light	7.85	35	16.5	616.45	1851.57	103.15	1976.73	2111.42
2	Ambient	light	7.92	35	16.5	513.62	1812.52	118.64	1949.49	2111.42
2	Ambient	light	7.93	35	16.5	500.28	1806.56	121.00	1945.42	2111.42
2	Ambient	light	7.95	35	16.5	474.53	1794.34	125.85	1937.12	2111.42
2	Ambient	light	7.95	35	16.5	474.53	1794.34	125.85	1937.12	2111.42
2	Enriched	light	7.92	35	16.5	513.62	1812.52	118.64	1949.49	2111.42
2	Enriched	light	7.93	35	16.5	500.28	1806.56	121.00	1945.42	2111.42
2	Enriched	light	8.09	35	16.5	325.16	1697.25	164.32	1873.17	2111.42
2	Enriched	light	7.97	35	16.5	449.98	1781.72	130.85	1928.63	2111.42
3	Ambient	light	8.04	35	16.5	372.80	1734.29	149.64	1897.24	2111.42

MFC	NH_4^+ treatment	cycle	pH_T	Salinity (‰)	Temperature (°C)	pCO_2 (µatm)	HCO_3^- (µmol/kg)	CO_3^{2-} (µmol/kg)	DIC (µmol/kg)	AT (µmol/kg)
3	Ambient	light	8.05	35	16.5	362.81	1727.10	152.49	1892.54	2111.42
3	Ambient	light	7.97	35	16.5	449.98	1781.72	130.85	1928.63	2111.42
3	Ambient	light	7.97	35	16.5	449.98	1781.72	130.85	1928.63	2111.42
3	Enriched	light	8.06	35	16.5	353.05	1719.80	155.39	1887.78	2111.42
3	Enriched	light	8.07	35	16.5	343.53	1712.39	158.32	1882.97	2111.42
3	Enriched	light	7.99	35	16.5	426.59	1768.68	136.01	1919.92	2111.42
3	Enriched	light	8.00	35	16.5	415.31	1762.01	138.66	1915.50	2111.42
1	Ambient	dark	7.69	35	16.5	926.19	1924.61	74.18	2031.85	2111.42
1	Ambient	dark	7.71	35	16.5	880.83	1916.62	77.35	2025.41	2111.42
1	Ambient	dark	7.62	35	16.5	1102.72	1950.32	63.98	2053.66	2111.42
1	Ambient	dark	7.69	35	16.5	926.19	1924.61	74.18	2031.85	2111.42
1	Enriched	dark	7.56	35	16.5	1278.69	1969.73	56.28	2071.64	2111.42
1	Enriched	dark	7.66	35	16.5	998.34	1936.06	69.64	2041.33	2111.42
1	Enriched	dark	7.65	35	16.5	1023.53	1939.73	68.18	2044.44	2111.42
1	Enriched	dark	7.66	35	16.5	998.34	1936.06	69.64	2041.33	2111.42
2	Ambient	dark	7.75	35	16.5	796.24	1899.69	84.07	2012.18	2111.42
2	Ambient	dark	7.76	35	16.5	776.30	1895.26	85.82	2008.79	2111.42
2	Ambient	dark	7.75	35	16.5	796.24	1899.69	84.07	2012.18	2111.42
2	Ambient	dark	7.75	35	16.5	796.24	1899.69	84.07	2012.18	2111.42
2	Enriched	dark	7.68	35	16.5	949.68	1928.50	72.64	2035.03	2111.42
2	Enriched	dark	7.75	35	16.5	796.24	1899.69	84.07	2012.18	2111.42
2	Enriched	dark	7.68	35	16.5	949.68	1928.50	72.64	2035.03	2111.42
2	Enriched	dark	7.71	35	16.5	880.83	1916.62	77.35	2025.41	2111.42
3	Ambient	dark	7.8	35	16.5	701.06	1876.71	93.18	1994.91	2111.42
3	Ambient	dark	7.81	35	16.5	683.33	1871.86	95.11	1991.35	2111.42
3	Ambient	dark	7.84	35	16.5	632.58	1856.78	101.09	1980.44	2111.42

MFC	NH_4^+ treatment	cycle	pH_T	Salinity (‰)	Temperature (°C)	pCO_2 (µatm)	HCO_3^- (µmol/kg)	CO_3^{2-} (µmol/kg)	DIC (µmol/kg)	AT (µmol/kg)
3	Ambient	dark	7.85	35	16.5	616.45	1851.57	103.15	1976.73	2111.42
3	Enriched	dark	7.79	35	16.5	719.21	1881.48	91.29	1998.44	2111.42
3	Enriched	dark	7.78	35	16.5	737.80	1886.16	89.44	2001.92	2111.42
3	Enriched	dark	7.82	35	16.5	666.02	1866.92	97.06	1987.76	2111.42
3	Enriched	dark	7.8	35	16.5	701.06	1876.71	93.18	1994.91	2111.42
1	Ambient	whole	7.81	35	16.5	683.33	1871.86	95.11	1991.35	2111.42
1	Ambient	whole	7.78	35	16.5	737.80	1886.16	89.44	2001.92	2111.42
1	Ambient	whole	7.7	35	16.5	903.25	1920.65	75.75	2028.64	2111.42
1	Ambient	whole	7.78	35	16.5	737.80	1886.16	89.44	2001.92	2111.42
1	Enriched	whole	7.69	35	16.5	926.19	1924.61	74.18	2031.85	2111.42
1	Enriched	whole	7.76	35	16.5	776.30	1895.26	85.82	2008.79	2111.42
1	Enriched	whole	7.76	35	16.5	776.30	1895.26	85.82	2008.79	2111.42
1	Enriched	whole	7.76	35	16.5	776.30	1895.26	85.82	2008.79	2111.42
2	Ambient	whole	7.84	35	16.5	632.58	1856.78	101.09	1980.44	2111.42
2	Ambient	whole	7.85	35	16.5	616.45	1851.57	103.15	1976.73	2111.42
2	Ambient	whole	7.86	35	16.5	600.70	1846.28	105.25	1972.97	2111.42
2	Ambient	whole	7.86	35	16.5	600.70	1846.28	105.25	1972.97	2111.42
2	Enriched	whole	7.81	35	16.5	683.33	1871.86	95.11	1991.35	2111.42
2	Enriched	whole	7.85	35	16.5	616.45	1851.57	103.15	1976.73	2111.42
2	Enriched	whole	7.88	35	16.5	570.28	1835.41	109.56	1965.32	2111.42
2	Enriched	whole	7.85	35	16.5	616.45	1851.57	103.15	1976.73	2111.42
3	Ambient	whole	7.92	35	16.5	513.62	1812.52	118.64	1949.49	2111.42
3	Ambient	whole	7.93	35	16.5	500.28	1806.56	121.00	1945.42	2111.42
3	Ambient	whole	7.92	35	16.5	513.62	1812.52	118.64	1949.49	2111.42
3	Ambient	whole	7.92	35	16.5	513.62	1812.52	118.64	1949.49	2111.42
3	Enriched	whole	7.93	35	16.5	500.28	1806.56	121.00	1945.42	2111.42

MFC	NH_4^+ treatment	cycle	pH_T	Salinity (‰)	Temperature (°C)	pCO_2 (µatm)	HCO_3^- (µmol/kg)	CO_3^{2-} (µmol/kg)	DIC (µmol/kg)	AT (µmol/kg)
3	Enriched	whole	7.94	35	16.5	487.25	1800.50	123.40	1941.29	2111.42
3	Enriched	whole	7.92	35	16.5	513.62	1812.52	118.64	1949.49	2111.42
3	Enriched	whole	7.91	35	16.5	527.28	1818.39	116.31	1953.52	2111.42
